# Draft Genome Sequences of Five Environmental Bacterial Isolates That Degrade Polyethylene Terephthalate Plastic

**DOI:** 10.1128/MRA.00237-19

**Published:** 2019-06-20

**Authors:** Rosa León-Zayas, Cameron Roberts, Morgan Vague, Jay L. Mellies

**Affiliations:** a Department of Biology, Willamette University, Salem, Oregon, USA; b Department of Biology, Reed College, Portland, Oregon, USA; Indiana University, Bloomington

## Abstract

Here, we report the annotated draft genome sequences of three Pseudomonas spp. and two Bacillus spp. that, as consortia, degrade polyethylene terephthalate plastic. Improved microbial degradation of plastic waste could help reduce the billions of metric tons of these materials that currently exist in our environment.

## ANNOUNCEMENT

**FIG 1:**
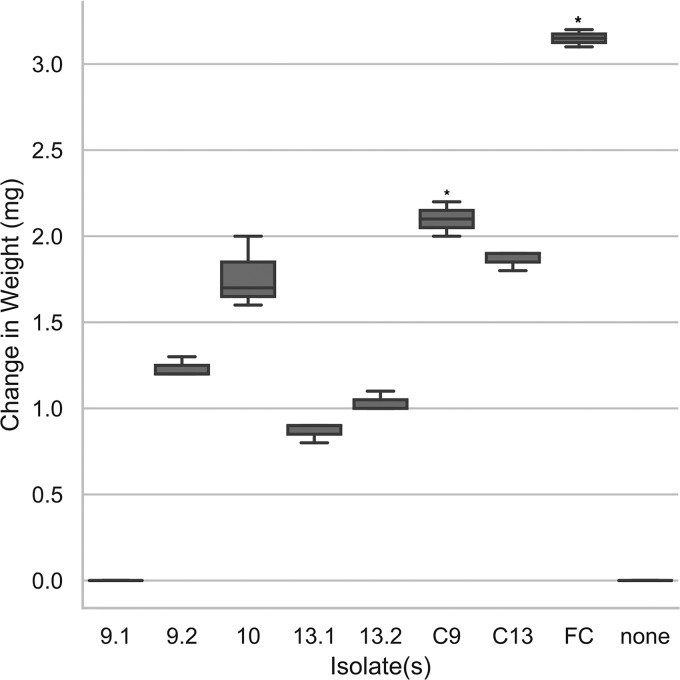
The full consortium, including all five strains, degrades PET to a greater extent than does any individual isolate or individual consortium after a 6-week incubation. Granular PET (Sigma-Aldrich, St. Louis, MO) was pretreated overnight with UV radiation (250 nm). Cultures of the five individual strains were grown overnight in LB broth and diluted to an optical density at 600 nm (OD_600_) of 1 to ensure that equal amounts of bacteria were added to each sample. Aliquots of each overnight culture were washed with phosphate-buffered saline (pH 7.4) twice, and equal volumes of bacteria were added to the appropriate 10-ml culture of LCFBM with 0.1 g total PET (starting OD_600_, 0.02). The samples were incubated at 30°C without shaking for 6 weeks. A negative control with UV-treated granular PET in medium without inoculation was kept under the same conditions. The granular PET weight loss from consortium 9 (least square means [LSM]_9_, 2.1 mg; standard deviation [SD], 0.05) treatment was statistically significantly greater than that with isolate 9.2 (*P* = 0.0002). The granular PET weight loss from the full consortium (FC) (LSM_FC_, 3.15 mg; SD, 0.07) treatment was statistically significantly greater than that for all isolates (*P* < 0.0001). Error bars indicate 1 SD.

Polyethylene terephthalate (PET) makes up a significant percentage of waste that accumulates in landfills and oceans, where it persists for decades or centuries. Seeking a method to aid in the accelerated decomposition of this material, we isolated five organisms that degrade PET. We predicted that bacteria that can degrade petroleum can potentially degrade chemically related plastics; thus, we obtained soil samples from locations polluted with petroleum products adjacent to Superfund sites near Houston, TX. Since lipases are enzymes associated with plastic degradation, we initially screened environmental isolates for lipase activity on rhodamine B agar plates, using olive oil as a source of long-chain fatty acids. Rhodamine B dye binds to free fatty acids (cleaved by a lipase) and glows when exposed to UV light at 365 nm. Thus, the presence of glowing halos around the colonies indicated lipase activity. Gram staining was used to monitor the purity of the cultures. In total, 192 colonies were screened after appearing to be lipase positive. One colony, named consortium 9, contained two strains, 9.1 and 9.2, while another colony, named consortium 13, contained two strains, 13.1 and 13.2. A single strain, 10, was also putatively positive for lipase activity. Description of the isolation of the strains can be found at https://www.ted.com/talks/morgan_vague_this_bacteria_eats_plastic. The consortia and the axenic strains were grown in liquid carbon-free basal medium (LCFBM) prepared with deionized water containing (per liter) 0.7 g of KH_2_PO_4_, 0.7 g of K_2_HPO_4_, 0.7 g of MgSO_4_·7H_2_O, 1.0 g of NH_4_NO_3_, 0.005 g of NaCl, 0.002 g of FeSO_4_·7H_2_O, 0.002 g of ZnSO_4_·7H_2_O, and 0.001 g of MnSO_4_·H_2_O (1). PET was introduced to LCFBM as a sole carbon source, and incubation with bacteria occurred at 30°C with shaking for 6 weeks. With UV pretreatment, all strains and consortia reduced the weight of the granular PET, except strain 9.1, which was unable to grow on the plastic as a sole carbon source (Fig. 1). However, consortium 9, containing strains 9.1 and 9.2, reduced the weight of the PET to a greater extent than did strain 9.2 alone (*P* = 0.0002). Interestingly, the full consortium (FC) containing all five strains reduced the weight of the granular PET more over the 6-week period than did any of the other individual strains or consortia (*P* < 0.0001). The 100-mg PET granule weighed 3.15 mg less after incubation with the full consortium, at an ∼3% reduction rate, and these results suggest that the strains can act synergistically to degrade PET plastic.

To identify the metabolic pathways and genes associated with PET degradation, we obtained genome sequences of the five strains. Briefly, bacteria were grown in lysogeny broth at 26°C overnight. DNA was extracted using the GenElute bacterial genomic DNA kit (MilliporeSigma, St. Louis, MO). For library preparation, performed at the Oregon State University (OSU) Center for Genome Research and Biocomputing, Illumina’s Nextera XT DNA sample prep kit (San Diego, CA) was used, following the manufacturer’s instructions. Sequencing was done on an Illumina MiSeq instrument with a run type of 150-bp paired-end fragments on a micro flow cell. The quality of the sequence fragments was assessed using FastQC (v0.11.5 [2]) and Trimmomatic (v0.36 [3]) for a quality standard of Q30 (LEADING:3 TRAILING:3 HEADCROP:10 SLIDINGWINDOW:4:30 MINLEN:36). High-quality sequence fragments (1,652,364 read average per sample) were then assembled using SPAdes (v3.13.0 [4]) with paired-end reads and high-quality singletons. The quality and genome metrics were analyzed using QUAST (v5) and are presented in Table 1 (5). The draft genome sizes range from 5,261,475 to 6,456,746 bp, and the GC content is 34.9% for the *Bacillus* draft genomes and 61.5% for the Pseudomonas draft genomes. Assemblies were annotated using Prokka (v1.13.3 [6]). An average of 5,557 protein-coding sequences were predicted for the draft genomes of our strains. Close relatives of 16S rRNA genes were as follows: for 9.1, Bacillus thuringiensis strain C15 (100% coverage/100% identity); for 9.2, *Pseudomonas* sp. B10 (100% coverage/99% identity); for 10, *Pseudomonas* sp. SWI36 (100% coverage/100% identity); for 13.1, Bacillus albus strain PFYN01 (100% coverage/100% identity); and for 13.2, *Pseudomonas* sp. SWI36 (100% coverage/100% identity). Preliminary metabolic comparisons using the KEGG database via BlastKOALA (7) have shown that the genomes share central carbohydrate metabolism and biosynthetic capabilities, such as synthesis of nucleotides and amino acids. The genomes also share genes associated with transport systems for simple and complex biomolecules. Each genome has numerous genes predicted to encode lipase-like enzymes. Interesting differences observed from these analyses include the potential for assimilatory sulfate reduction in three out of the five genomes (9.2, 10, and 13.2). Two other genomes possess complete pathways for dissimilatory nitrate reduction (9.1 and 13.1).

**TABLE 1:** Draft genome assembly and annotation metrics

Strain	No. of high-quality reads	Genome size (bp)	No. of contigs >1 kb	GC content (%)	*N*_50_ value (bp)	Largest contig (bp)	Top BlastN 16S rRNA hit	No. of predicted proteins (Prokka)	BioSample accession no.
9.1	1,948,613	5,261,475	139	35.0	103,322	252,489	Bacillus thuringiensis strain C15	5,275	SAMN10824115
9.2	1,258,720	6,215,199	118	60.7	105,169	354,110	*Pseudomonas* sp. B10	5,565	SAMN10824116
10	1,582,339	5,786,271	214	61.9	50,964	247,856	*Pseudomonas* sp. SWI36	5,186	SAMN10824117
13.1	1,657,136	6,456,746	477	34.9	31,034	166,467	Bacillus albus strain PFYN01	6,566	SAMN10824118
13.2	1,815,014	5,790,691	208	61.9	50,542	258,222	*Pseudomonas* sp. SWI36	5,193	SAMN10824119

### Data availability.

The DNA sequences and genome assemblies have been deposited in GenBank under BioProject number PRJNA517285 and the following SRA and BioSample accession numbers, respectively: SRX5623359 and SAMN10824115 for strain 9.1, SRX5623360 and SAMN10824116 for strain 9.2, SRX5623357 and SAMN10824117 for strain 10, SRX5623358 and SAMN10824118 for strain 13.1, and SRX5623356 and SAMN10824119 for strain 13.2.
